# Supplementary material for: Whole-genome sequencing analysis of the cardiometabolic proteome
Source: Nat Commun. 2020 Dec 10;11:6336. doi: 10.1038/s41467-020-20079-2 (PMC7729872; doi:10.1038/s41467-020-20079-2)
Supplement: Supplementary file 1 — Supplementary Information [file 41467_2020_20079_MOESM1_ESM.pdf]

Supplementary Information

Supplementary Figures

Supplementary Figure 1: *cis*-association within the *CTSH* gene. Independent variants as defined by COJO[1] are highlighted by crosses.

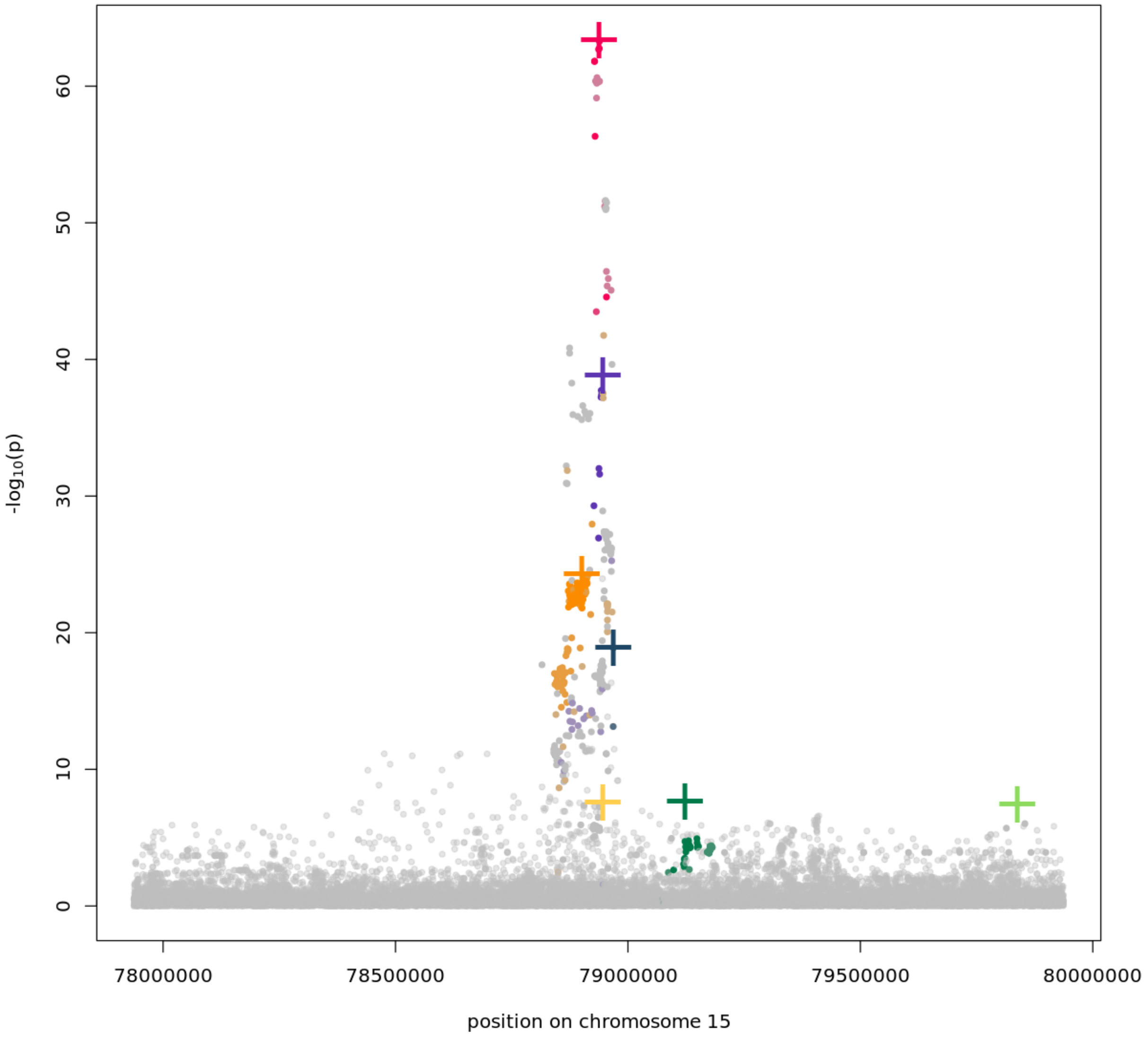

Supplementary Figure 2: Effect size according to log-MAF for independent variants at pQTLs discovered in this study. Question marks denote variants not present in the replication dataset and for which no LD-based proxy (r^2>0.8) could be found, crosses indicate variants that were tested but did not pass the replication significance threshold. Error bars denote standard errors.

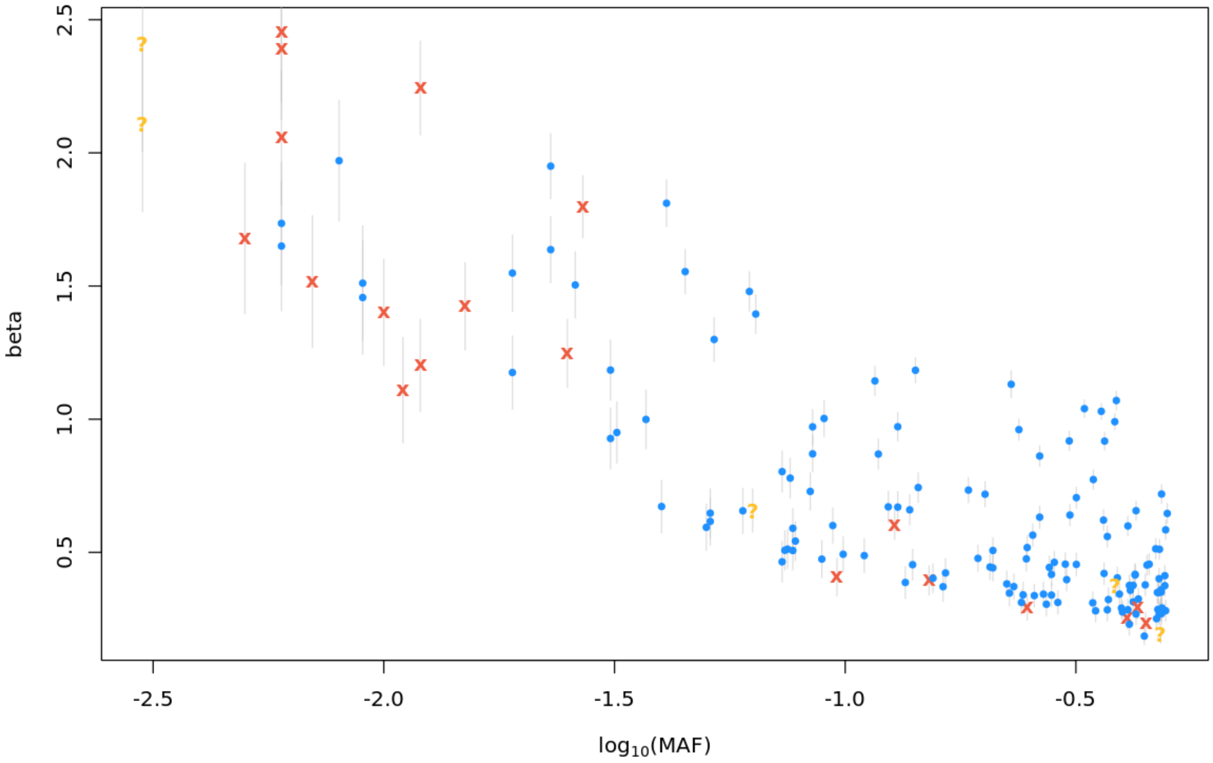

Supplementary Figure 3: Variance explained in proteomic traits compared with 37 non-proteomic traits (Supplementary Data 15) in the same cohort using the same association protocol. Whiskers extend to 1.5 times the interquartile range denoted by the boxes. The bold line denotes the median.

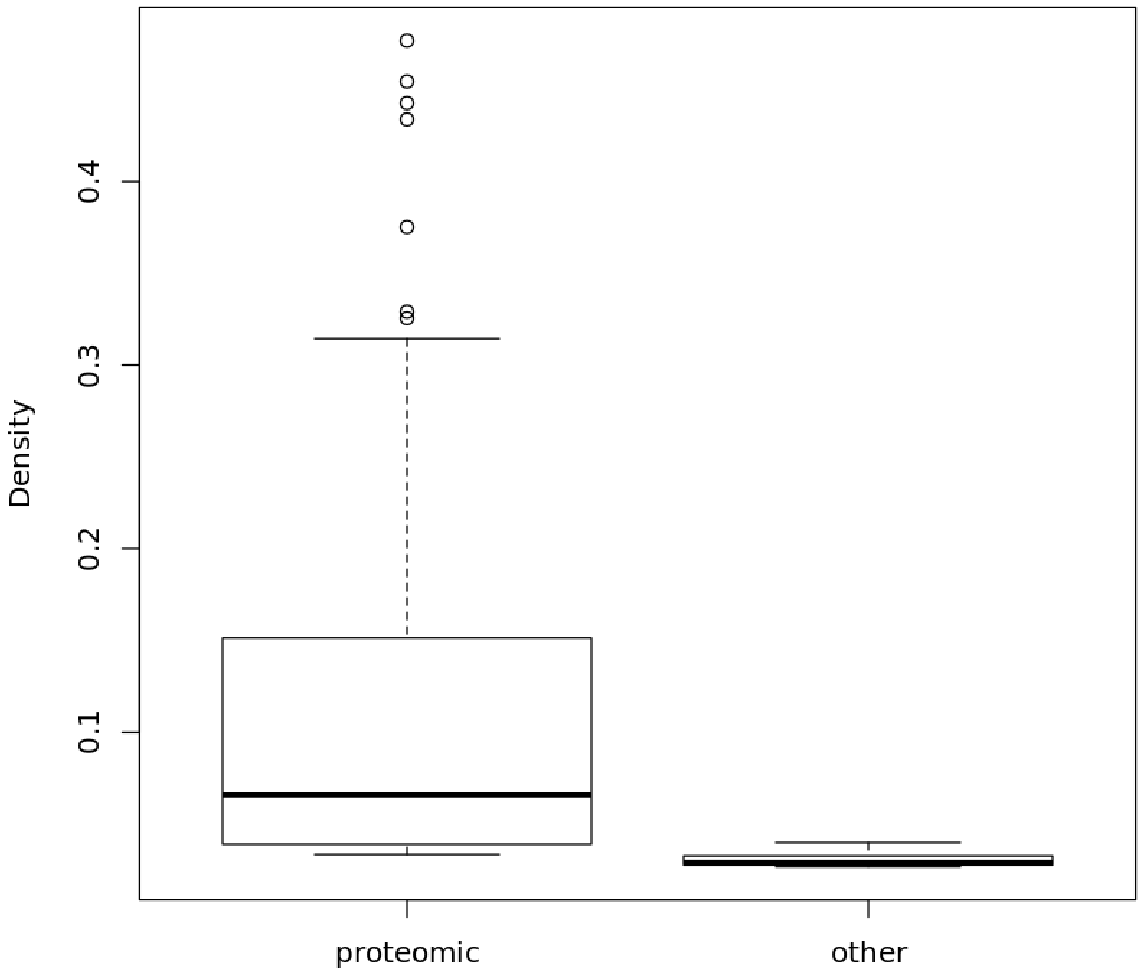

Supplementary Figure 4: Significant burden associations across all analyses. Numbers and colour scale represent significance on the -log10 scale. Columns are variant selection methods (see Methods), rows are the proteins for which these significant *cis*-acting associations were found.

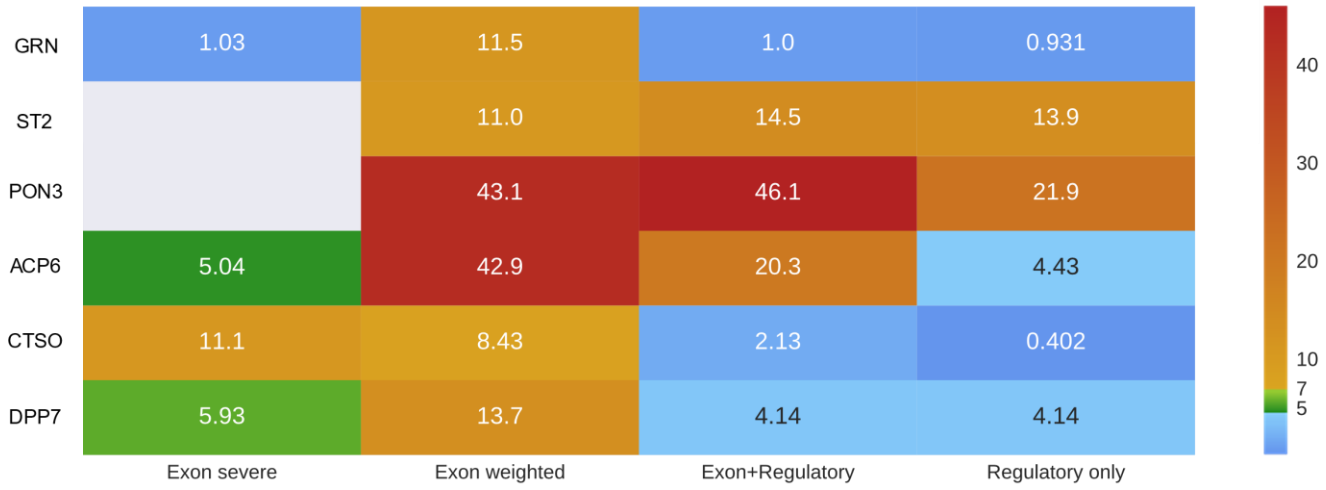

Supplementary Figure 5. Elastic net model of high cholesterol. Top left: Precision-recall curves for models with different values of alpha on the holdout set. The ridge model curve (alpha=0) is coloured by lambda value according to the scale on the right of the plot. All other models are drawn on a purple to yellow scale, with yellow corresponding to alpha=0.1 and purple to alpha=1. Top right: Area under the curve for the optimal lambdas + 1 standard error, according to alpha, for predictions on the holdout set. Bottom left: Mean-square error estimate and standard error according to lambda, for alpha=0.1, on the training set, over 5 repetitions of 5-fold cross validations (n=25). The two vertical lines indicate the minimal lambda and the minimal lambda + 1 standard error, respectively. Bottom left: Coefficient trajectory as a function of log(lambda) for the training set, with the top 4 coefficients in absolute value highlighted. The model coefficients fit on the entire dataset are given in the Supplementary Note.

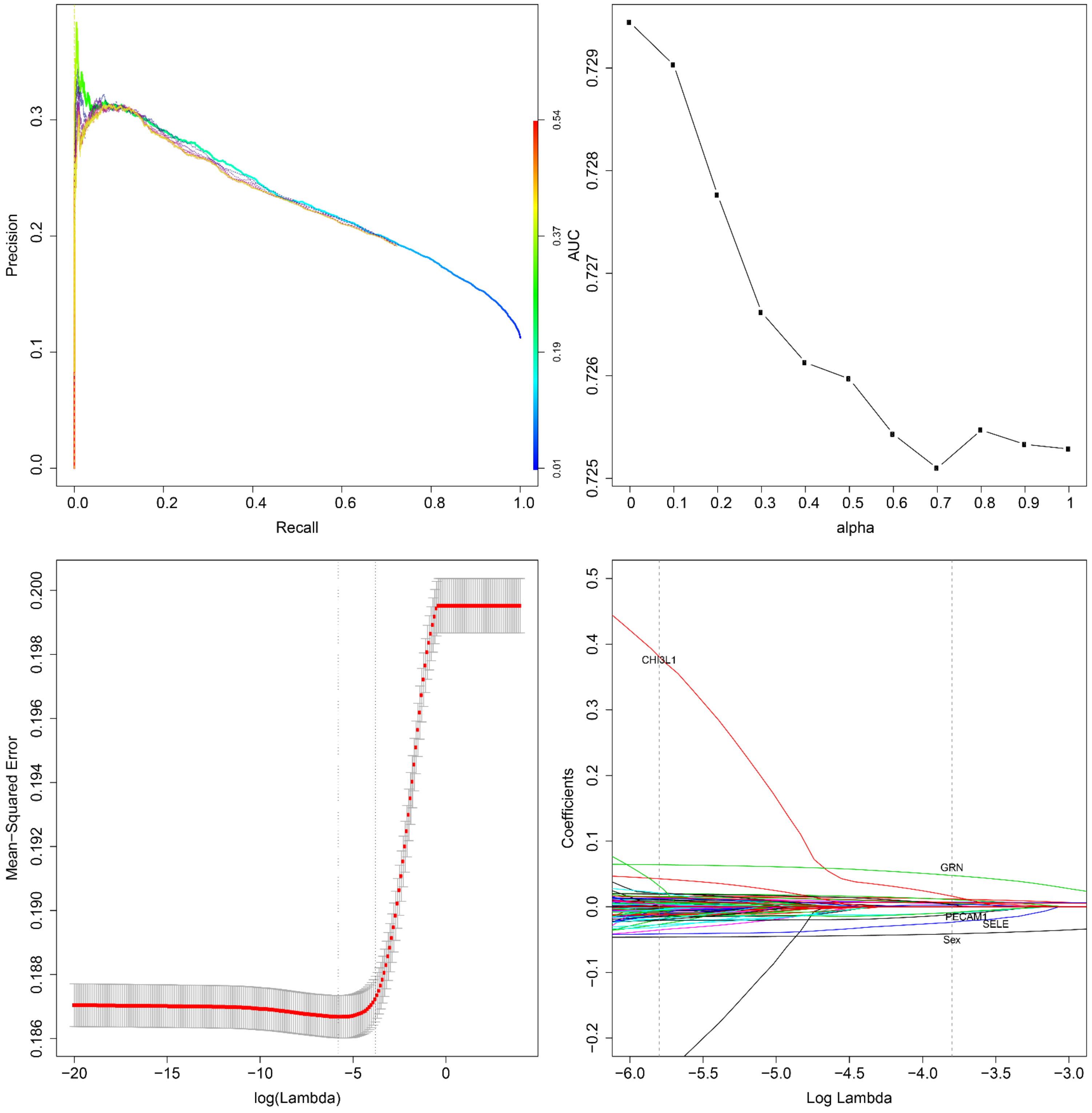

Supplementary Figure 6: Evaluation of effective number of tests and significance thresholds. Reporting single-point (a,d), sliding-window rare variant burden test (b, e) and both (c, f), using two different simulation models (a, b, c and d, e, f, respectively), on chromosome 11, across 1,000 simulations.

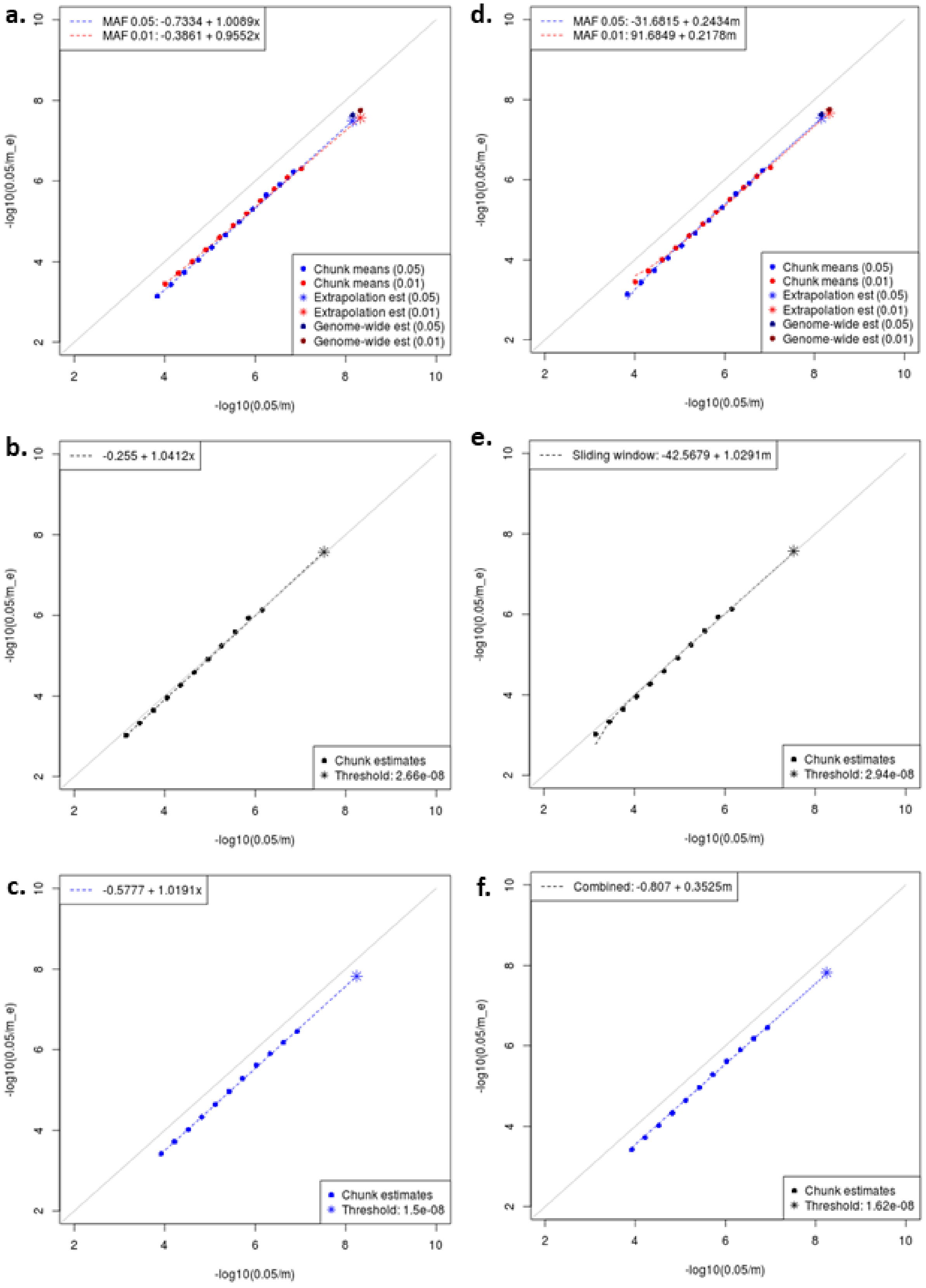

Supplementary Figure 7: Distance to gene boundary as defined by Ensembl REST API for *cis* independent variants. Points are arranged semi-randomly on the x axis to visualise the distribution of distances.

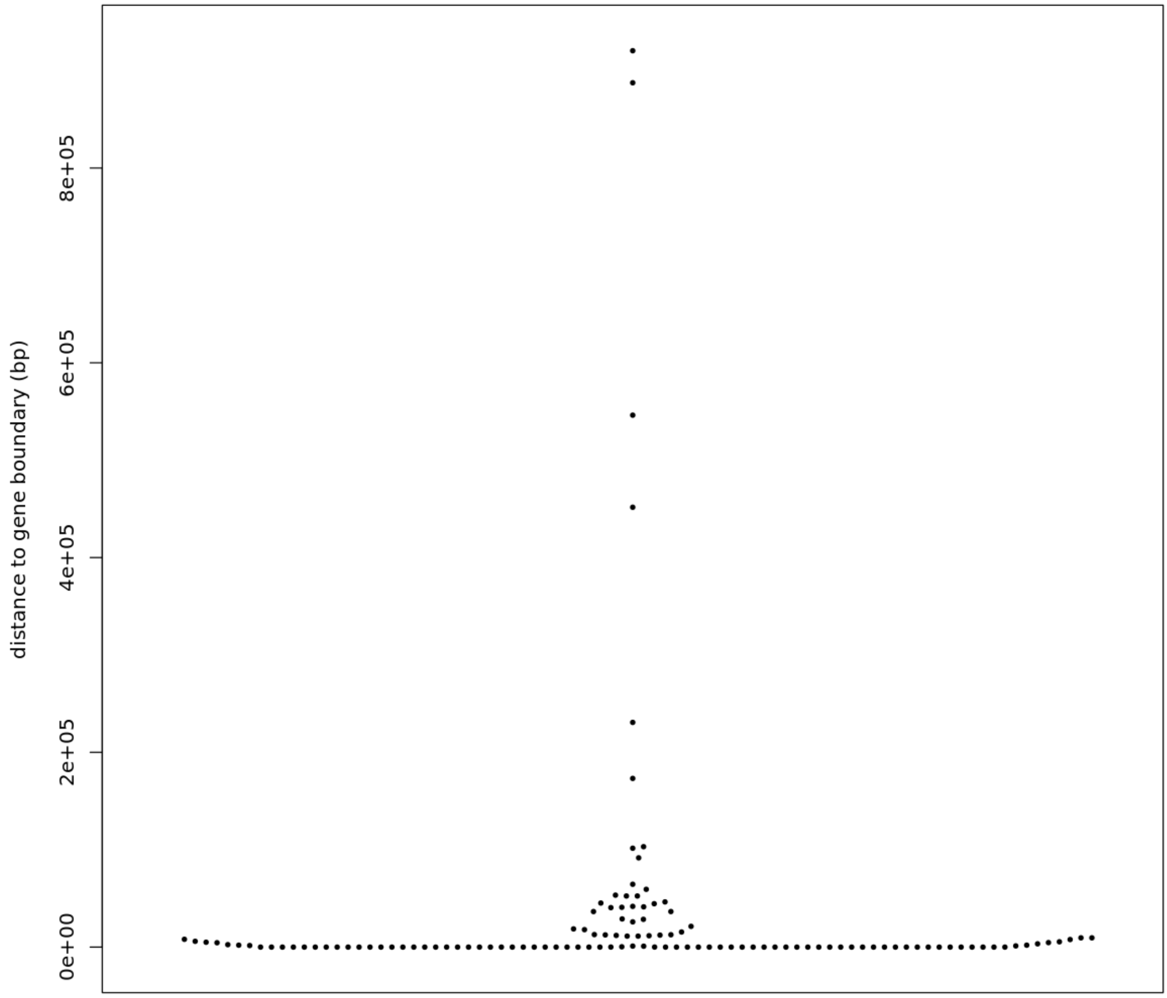

Supplementary Notes

Supplementary Note 1: Overview of single-point cis signals

117 (90%) of 131 variants reproducibly associated in the MANOLIS cohort are common (minor allele frequency (MAF) >5%), 13 are low-frequency (MAF 1-5%) and 1 is rare (MAF<1%) (Figure 2). One hundred of the associated variants (76%) are located within 1Mb of the gene encoding the respective protein (i.e. in *cis*-pQTLs), and 31 (24%) are in *trans*-pQTLs (Figure 2). 57 *cis*-associated variants are located within the boundaries of the respective gene, and the remaining 43 are at a median distance of 13.8kb (max. 920 kb) (Supplementary Figure 7).

Supplementary Note 2: Joint model of high cholesterol using CHI3L1 and PECAM1

To quantify cumulative contributions of predictive proteins to hypercholesterolemia risk, we perform a joint logistic model of high cholesterol using GRN, CHI3L1 and PECAM1 scores, including clinical and genetic covariates as predictors. The GRN score is driven by a well-known association at the *SORT1* locus, and the PECAM1 score is driven by a known association near the *ABO* gene[2,3]. The full model is reported below:

```
Call:
glm(formula = "high_cholesterol~.", family = binomial(link = "logit"),
    data = m)

Deviance Residuals:
    Min       1Q   Median       3Q      Max
-1.6414  -0.5371  -0.3833  -0.2555   3.1473

Coefficients:
                                      Estimate Std. Error  z value Pr(>|z|)
(Intercept)                         -9.0831972  0.0574584 -158.083  < 2e-16 ***
Sex2                                -0.5171506  0.0107463  -48.124  < 2e-16 ***
Smoking_status1                      0.1442364  0.0112892   12.777  < 2e-16 ***
Smoking_status2                      0.2427965  0.0185230   13.108  < 2e-16 ***
Age_when_attended_assessment_centre  0.0900875  0.0007782  115.762  < 2e-16 ***
Qualifications2                      0.0983272  0.0171074    5.748 9.05e-09 ***
Qualifications3                      0.1647759  0.0134793   12.224  < 2e-16 ***
Qualifications4                      0.2183363  0.0238748    9.145  < 2e-16 ***
Qualifications5                      0.1876037  0.0186386   10.065  < 2e-16 ***
Qualifications6                      0.1740369  0.0204804    8.498  < 2e-16 ***
PC1                                  0.0016187  0.0001049   15.433  < 2e-16 ***
PC2                                 -0.0030686  0.0001921  -15.977  < 2e-16 ***
PC3                                  0.0057971  0.0003399   17.053  < 2e-16 ***
PC4                                 -0.0016641  0.0004544   -3.662  0.00025 ***
PC5                                  0.0004729  0.0007103    0.666  0.50551
PC6                                 -0.0004193  0.0011021   -0.380  0.70359
PC7                                 -0.0003830  0.0010368   -0.369  0.71180
PC8                                 -0.0019398  0.0011052   -1.755  0.07924 .
PC9                                  0.0007471  0.0011426    0.654  0.51318
PC10                                 0.0061919  0.0012054    5.137 2.79e-07 ***
BMI                                  0.0706469  0.0010486   67.374  < 2e-16 ***
PECAM1                              -0.7194621  0.0784022   -9.177  < 2e-16 ***
CHI3L1                               0.7271593  0.1181912    6.152 7.63e-10 ***
GRN                                  0.7638145  0.0360353   21.196  < 2e-16 ***
---
Signif. codes:  0 '***' 0.001 '**' 0.01 '*' 0.05 '.' 0.1 ' ' 1

(Dispersion parameter for binomial family taken to be 1)

    Null deviance: 278033  on 395511  degrees of freedom
Residual deviance: 251654  on 395488  degrees of freedom
  (91897 observations deleted due to missingness)
AIC: 251702

Number of Fisher Scoring iterations: 5
```

This model produced an AUC of 0.7297. All three scores significantly contributed to the model. To eliminate the effect of the well-known single-point signals, we exclude 1Mb regions around *ABO* and *CELSR2-SORT1* from the score calculation and rerun the model, which is given below:

```
Call:
glm(formula = "high_cholesterol~.", family = binomial(link = "logit"), 
    data = m)

Deviance Residuals: 
    Min       1Q   Median       3Q      Max  
-1.6107  -0.5383  -0.3848  -0.2574   3.1255  

Coefficients:
                                  Estimate Std. Error  z value Pr(>|z|)    
(Intercept)                     -9.1238641  0.0575307 -158.591  < 2e-16 ***
Sex2                            -0.5159981  0.0107369  -48.058  < 2e-16 ***
Smoking_status1                  0.1428085  0.0112786   12.662  < 2e-16 ***
Smoking_status2                  0.2408256  0.0185052   13.014  < 2e-16 ***
Age_when_attended_assessment_centre  0.0899196  0.0007774  115.665  < 2e-16 ***
Qualifications2                  0.0988930  0.0170929    5.786 7.22e-09 ***
Qualifications3                  0.1649018  0.0134672   12.245  < 2e-16 ***
Qualifications4                  0.2202663  0.0238549    9.234  < 2e-16 ***
Qualifications5                  0.1888843  0.0186188   10.145  < 2e-16 ***
Qualifications6                  0.1752382  0.0204611    8.564  < 2e-16 ***
PC1                              0.0010894  0.0001030   10.577  < 2e-16 ***
PC2                             -0.0034572  0.0001913  -18.073  < 2e-16 ***
PC3                              0.0055507  0.0003407   16.294  < 2e-16 ***
PC4                             -0.0022321  0.0004534   -4.923 8.53e-07 ***
PC5                             -0.0001158  0.0007084   -0.163   0.8701    
PC6                             -0.0005177  0.0010989   -0.471   0.6376    
PC7                             -0.0001771  0.0010356   -0.171   0.8642    
PC8                             -0.0019557  0.0011038   -1.772   0.0765 .  
PC9                              0.0004376  0.0011404    0.384   0.7012    
PC10                             0.0062099  0.0012040    5.158 2.50e-07 ***
BMI                              0.0705330  0.0010474   67.342  < 2e-16 ***
PECAM1                          -0.0388835  0.0763970   -0.509   0.6108    
CHI3L1                           0.7254305  0.1180929    6.143 8.10e-10 ***
GRN                              0.0295028  0.0585299    0.504   0.6142    
---
Signif. codes:  0 '***' 0.001 '**' 0.01 '*' 0.05 '.' 0.1 ' ' 1

(Dispersion parameter for binomial family taken to be 1)

    Null deviance: 278033  on 395511  degrees of freedom
Residual deviance: 252197  on 395488  degrees of freedom
  (91897 observations deleted due to missingness)
AIC: 252245

Number of Fisher Scoring iterations: 5
```

The AUC is 0.7275, which is the same as for the model below, fully excluding PECAM1 and GRN scores:

```
Call:
glm(formula = "high_cholesterol~.", family = binomial(link = "logit"), 
    data = m)

Deviance Residuals: 
    Min       1Q   Median       3Q      Max  
-1.6108  -0.5383  -0.3848  -0.2574   3.1255  

Coefficients:
                                  Estimate Std. Error  z value Pr(>|z|)    
(Intercept)                     -9.1239355  0.0571687 -159.597  < 2e-16 ***
Sex2                            -0.5160183  0.0107367  -48.061  < 2e-16 ***
Smoking_status1                  0.1428000  0.0112786   12.661  < 2e-16 ***
```

```
Smoking_status2                     0.2408163  0.0185051   13.014  < 2e-16 ***
Age_when_attended_assessment_centre  0.0899201  0.0007774  115.666  < 2e-16 ***
Qualifications2                     0.0989089  0.0170927    5.787 7.18e-09 ***
Qualifications3                     0.1648949  0.0134671   12.244  < 2e-16 ***
Qualifications4                     0.2202643  0.0238549    9.234  < 2e-16 ***
Qualifications5                     0.1888697  0.0186186   10.144  < 2e-16 ***
Qualifications6                     0.1752434  0.0204609    8.565  < 2e-16 ***
PC1                                 0.0010921  0.0001021   10.699  < 2e-16 ***
PC2                                -0.0034577  0.0001911  -18.090  < 2e-16 ***
PC3                                 0.0055592  0.0003397   16.364  < 2e-16 ***
PC4                                -0.0022385  0.0004533   -4.938 7.88e-07 ***
PC5                                -0.0001143  0.0007084   -0.161    0.872    
PC6                                -0.0005177  0.0010990   -0.471    0.638    
PC7                                -0.0001706  0.0010355   -0.165    0.869    
PC8                                -0.0019518  0.0011037   -1.768    0.077 .  
PC9                                 0.0004448  0.0011403    0.390    0.696    
PC10                                0.0062099  0.0012040    5.158 2.50e-07 ***
BMI                                 0.0705311  0.0010474   67.341  < 2e-16 ***
CHI3L1                              0.7259139  0.1180905    6.147 7.89e-10 ***
---
Signif. codes:  0 '***' 0.001 '**' 0.01 '*' 0.05 '.' 0.1 ' ' 1

(Dispersion parameter for binomial family taken to be 1)

    Null deviance: 278033  on 395511  degrees of freedom
Residual deviance: 252197  on 395490  degrees of freedom
  (91897 observations deleted due to missingness)
AIC: 252241

Number of Fisher Scoring iterations: 5
```

Finally, the null model excluding any score is given below, with an AUC of 0.7273:

```
Call:
glm(formula = "high_cholesterol~.", family = binomial(link = "logit"), 
    data = m[, -c("PECAM1", "CHI3L1"), with = F])

Deviance Residuals: 
    Min       1Q   Median       3Q      Max  
-1.6277  -0.5384  -0.3851  -0.2575   3.1026  

Coefficients:
                                     Estimate Std. Error  z value Pr(>|z|)    
(Intercept)                        -9.1479772  0.0570438 -160.368  < 2e-16 ***
Sex2                               -0.5159580  0.0107361  -48.058  < 2e-16 ***
Smoking_status1                     0.1428811  0.0112779   12.669  < 2e-16 ***
Smoking_status2                     0.2407488  0.0185043   13.010  < 2e-16 ***
Age_when_attended_assessment_centre  0.0899048  0.0007774  115.654  < 2e-16 ***
Qualifications2                     0.0990247  0.0170914    5.794 6.88e-09 ***
Qualifications3                     0.1648836  0.0134662   12.244  < 2e-16 ***
Qualifications4                     0.2199415  0.0238540    9.220  < 2e-16 ***
Qualifications5                     0.1889738  0.0186179   10.150  < 2e-16 ***
Qualifications6                     0.1753056  0.0204601    8.568  < 2e-16 ***
PC1                                 0.0011130  0.0001020   10.910  < 2e-16 ***
PC2                                -0.0034758  0.0001911  -18.189  < 2e-16 ***
PC3                                 0.0055479  0.0003397   16.333  < 2e-16 ***
PC4                                -0.0023560  0.0004529   -5.203 1.97e-07 ***
PC5                                -0.0001256  0.0007084   -0.177   0.8592    
PC6                                -0.0005892  0.0010996   -0.536   0.5921    
PC7                                -0.0001620  0.0010354   -0.156   0.8757    
PC8                                -0.0020606  0.0011037   -1.867   0.0619 .  
PC9                                 0.0004355  0.0011402    0.382   0.7025    
PC10                                0.0062708  0.0012040    5.209 1.90e-07 ***
BMI                                 0.0704985  0.0010472   67.320  < 2e-16 ***
---
Signif. codes:  0 '***' 0.001 '**' 0.01 '*' 0.05 '.' 0.1 ' ' 1

(Dispersion parameter for binomial family taken to be 1)
```

```
    Null deviance: 278033  on 395511  degrees of freedom
Residual deviance: 252235  on 395491  degrees of freedom
  (91897 observations deleted due to missingness)
AIC: 252277

Number of Fisher Scoring iterations: 5
```

Likelihood ratio tests are computed using the lrtest function from the lmtest R package, comparing the first and second model to the null model. AUCs are computed using the roc function from the pROC R package, and DeLong's AUC test is computed using the roc.test function from the same package. The bootstrapping test for correlated ROC curves gives similar results.

Supplementary Note 3: Additional notable pQTL signals not described in the text

The *PLAUR* missense variant rs4760 is associated with decreased levels of TNFRSF10C (TNF receptor superfamily member 10c) (MAF=13%, β=-0.96, σ=0.054, P=7.31x10^{-52}). *PLAUR* codes for the urokinase receptor (uPAR). Urokinase is an essential thrombolytic agent and acts as an invasion-promoting protein in several types of cancer[4] through blocking efferocytosis and phagocytosis, notably in apoptotic cardiocytes[5]. This variant has previously been associated with decreased levels of the TRAIL apoptosis-inducing ligand[6], TNFSF10C. TRAIL has been shown to induce overexpression of urokinase, and uPAR acts as a "don't eat me" signal for apoptotic cells. This *trans*-pQTL finding could indicate that impairment of the urokinase receptor is linked to an oversensitivity to TRAIL signalling, leading to decreased levels of both TRAIL and its receptor.

rs10886430, an intronic *GRK5* variant, is associated with decreased CCL17 levels (MAF=9.9%, β=-0.493, σ=0.0656, P=3.72x10^{-13}). CCL17 restrains regulatory T cell homeostasis to promote atherosclerosis through binding to CCR4 and other receptors[7]. Acting through G protein-coupled chemokine receptors, ACKR1 ligands such as CCL17 can induce activation and migration of leucocyte subsets into the vessel wall, and play a pathogenic role during atherosclerosis development[8]. *GRK5* codes for a G protein-coupled receptor kinase, which desensitises activated G protein-coupled receptors through phosphorylation and subsequent binding of arrestin. This *trans*-pQTL finding could indicate a phosphorylation activity of GRK5 on one of the G-coupled receptors of CCL17 such as CCR4 or CCR8. rs10886430 is not a significant eQTL for any gene in any tissue[9].

rs144846334, an intergenic variant upstream of *SLC10A2*, is associated with decreased levels of EPCAM (β=-0.779, σ=0.0738, MAF=7.6%, P=3.52x10^{-23}, *trans*-pQTL). rs144846334 is in strong LD (r^2>0.8) with the *SLC10A2* missense variant rs56398830 (β=-0.77, σ=0.0730, P=5.42x10^{-23}). *SLC10A2* plays a key role in sodium-dependent intestinal bile salt reuptake, and variants in this gene have been associated with numerous phenotypes such as gallbladder diseases, venous thromboembolism, LDL and HDL cholesterol. *SLC10A2* knockout mice have decreased intestinal cholesterol absorption, abnormal bile salt levels and steatorrhea. Loss of *EPCAM* function, is causal for congenital tufting enteropathy[10], a severe sodium-losing diarrheal disorder presenting in the neonatal period.

rs1309620228, a start lost variant (MAF=0.2%, β=-2.21, σ=0.410, P=1.21x10^{-7}), and rs556026695, a splice donor variant (MAF=0.2%, β=-2.36, σ=0.457, P=3.63x10^{-7}) drive a *cis*-RV-pQTL for CTSO (P=7.94x10^{-12}). Both variants are present at much lower frequencies in cosmopolitan populations (MAC=1 in TOPMed for rs1309620228; MAC=1 in gnomAD and TOPMed for rs556026695). A third variant, the splice region variant rs763411023, is included but its contribution to the burden is small (P=0.066). The *CTSO* gene codes for cathepsin O, a cysteine protease with unclear function. Cathepsin O is ubiquitously expressed, and is involved in normal cellular protein degradation and turnover[11]. In mice, mutations in the *Ctso* gene have been associated with decreased bilirubin and aspartate

transaminase levels. rs11722604, an intronic *CTSO* variant, has previously been associated with increased adiponectin in an East Asian cohort. This rare variant burden signal replicates in Pomak (P=4.01x10^{-24} in the exon weighted analysis), and is entirely driven by the missense variant rs1013059201.

Supplementary Note 4: Additional analyses for variant prioritisation and consequence evaluation

Colocalisation testing for eQTL overlap and pheWAS analysis

We perform colocalisation testing with eQTL data from the GTEX database[12]. First, for every signal, regions are extended 1Mb either side of every independent variant, and associations are conditioned on every other variant in the peak using GEMMA. For *cis* signals, expression information for the *cis* gene is extracted from the GTEX database over the same region. For *trans* signals, expression information is restricted to all genes located within a 2Mb region surrounding the variant. Then, for every variant/gene pair, we perform colocalisation testing using the fast.coloc function from the gtx R package (https://github.com/tobyjohnson/gtx). We use the commonly chosen value of 0.8 as a posterior threshold to declare colocalization[13], and default values of 1x10^{-4}, with standard deviation of 1, for the prior probability of a variant to be causal for either trait, and 1x10^{-5}, with standard deviation of 1, for the prior probability of a variant to be causal for both traits. To account for multiple independent variants at the same locus, we perform conditional analysis on all independent variants except the one under consideration, and use these results as input for the colocalisation analysis. 60 (49%) independent variants in 45 (57%) *cis*-pQTLs co-localise with an expression quantitative trait locus (eQTL; identified in GTEx[9]) for the cis gene, in keeping with previous estimates for the proportion of pQTLs exerting their effects through transcriptional mechanisms[14]. In addition, we find that 29 (70%) *trans* signals co-localise with eQTLs for at least one gene in their vicinity (+/-1Mb).

We apply the same procedure to PhenoScanner data[15], where eQTL data is replaced by the output of the PhenoScanner python utility 500kb either side of every independent variant. The colocalisation procedure is then repeated for every phenotype where at least one association is present in the region. Because such pheWAS results do not necessarily report weakly associated SNVs, the number of pheWAS variants to be colocalised can be small. We therefore modify the colocalisation script to handle the case where only one variant is present per phenotype. 82% of all signals co-localised with previous phenotypic associations (Supplementary Data 16). The proportion was similar for *trans* and *cis* loci, with 30 out of 37 co-localising with previous association signals in the latter case. As a proof of concept, we recapitulate strong evidence for colocalization of a progranulin-lowering trans-pQTL in the *SORT1-CELSR2* locus with increased risk of elevated cholesterol, angina pectoris, ischaemic heart disease and other cardio-damaging phenotypes. *trans*-pQTL for all the six proteins associated with an ABO region variant also colocalise with a range of blood and cardiovascular phenotypes, in line with a large body of evidence linking the ABO blood group with multiple diseases.

Drug Target evaluation

For evaluating whether associated genes were drug targets, we used the OpenTargets[16] and DrugBank[17] databases. We accessed OpenTargets using the OpenTarget API. We converted the DrugBank XML file to flat files using the dbparser R package, and performed gene name matching using the USCS Gene Info database, downloaded May 6, 2019. 8 of the proteins for which a signal

was detected at study-wide significance were targeted by drugs according to OpenTargets. This was true for 39 proteins when queried against the DrugBank database (Supplementary Data 17).

Mouse models

We use the Ensembl REST API to extract mouse orthologs for all of the 109 genes whose protein for which genetic associations were found in our study. According to the IMPC API, KO experiments were reported for 26 of these orthologs, 18 of these having phenotypes associated with a p-value smaller than $1x10^{-4}$ (Supplementary Data 18).

Supplementary Note 5: Additional models produced by PheCode analysis

In addition to the associations discovered using ICD-10 codes alone, using PheCode as phenotypes produces several more associations, as detailed in Supplementary Data 12. In the paragraphs below, we demonstrate that these associations are all spurious, being caused by instabilities in coefficient values between the correlated predicted scores.

LRP11

We first examine LRP11, since it is constructed at a very low P-value and every other successful score has been sparse in our analysis.

First, we examine the original model:

```
Call:
glm(formula = Phecode_411.8 ~ ., family = "binomial", data = d)

Deviance Residuals:
    Min       1Q   Median       3Q      Max
-1.2733  -0.3186  -0.2118  -0.1363   3.6495

Coefficients:
                                    Estimate Std. Error  z value Pr(>|z|)
(Intercept)                       -1.118e+01  1.088e-01 -102.714  < 2e-16 ***
Sex2                              -1.094e+00  2.046e-02  -53.493  < 2e-16 ***
Smoking_status1                    3.171e-01  1.993e-02   15.909  < 2e-16 ***
Smoking_status2                    6.111e-01  2.981e-02   20.497  < 2e-16 ***
Age_when_attended_assessment_centre  1.019e-01  1.476e-03   69.017  < 2e-16 ***
Qualifications2                    1.969e-01  3.092e-02    6.369 1.90e-10 ***
Qualifications3                    2.794e-01  2.407e-02   11.608  < 2e-16 ***
Qualifications4                    4.370e-01  4.166e-02   10.491  < 2e-16 ***
Qualifications5                    3.979e-01  2.982e-02   13.343  < 2e-16 ***
Qualifications6                    2.988e-01  3.477e-02    8.591  < 2e-16 ***
PC1                                1.232e-03  2.068e-04    5.956 2.59e-09 ***
PC2                               -4.597e-03  3.044e-04  -15.099  < 2e-16 ***
BMI                                7.492e-02  1.785e-03   41.963  < 2e-16 ***
META.LRP11.7.45e11                -1.618e+00  3.657e-01   -4.425 9.64e-06 ***
META.LRP11.1.97e7                  2.109e+00  6.773e-01    3.114  0.00184 **
META.LRP11.9.70e7                 -4.409e-02  6.211e-01   -0.071  0.94341
---
Signif. codes:  0 '***' 0.001 '**' 0.01 '*' 0.05 '.' 0.1 ' ' 1

(Dispersion parameter for binomial family taken to be 1)

    Null deviance: 109932  on 318958  degrees of freedom
Residual deviance:  96694  on 318943  degrees of freedom
  (81924 observations deleted due to missingness)
AIC: 96726

Number of Fisher Scoring iterations: 7
```

Then, we perform the same analysis with only the sparsest (and most significant) score:

```
Call:
glm(formula = Phecode_411.8 ~ ., family = "binomial", data = d)

Deviance Residuals:
    Min       1Q   Median       3Q      Max
-1.2709  -0.3186  -0.2119  -0.1363   3.6507

Coefficients:
                                      Estimate Std. Error  z value Pr(>|z|)
(Intercept)                         -1.118e+01  1.088e-01 -102.770  < 2e-16 ***
Sex2                                -1.095e+00  2.046e-02  -53.518  < 2e-16 ***
Smoking_status1                      3.170e-01  1.993e-02   15.905  < 2e-16 ***
Smoking_status2                      6.112e-01  2.981e-02   20.504  < 2e-16 ***
Age_when_attended_assessment_centre  1.019e-01  1.476e-03   69.004  < 2e-16 ***
Qualifications2                      1.971e-01  3.092e-02    6.375 1.83e-10 ***
Qualifications3                      2.792e-01  2.407e-02   11.602  < 2e-16 ***
Qualifications4                      4.365e-01  4.166e-02   10.477  < 2e-16 ***
Qualifications5                      3.971e-01  2.982e-02   13.316  < 2e-16 ***
Qualifications6                      2.990e-01  3.477e-02    8.598  < 2e-16 ***
PC1                                  1.216e-03  2.067e-04    5.886 3.96e-09 ***
PC2                                 -4.707e-03  3.016e-04  -15.607  < 2e-16 ***
BMI                                  7.491e-02  1.785e-03   41.959  < 2e-16 ***
META.LRP11                          -1.534e-01  1.032e-01   -1.486    0.137
---
Signif. codes:  0 '***' 0.001 '**' 0.01 '*' 0.05 '.' 0.1 ' ' 1

(Dispersion parameter for binomial family taken to be 1)

    Null deviance: 109932  on 318958  degrees of freedom
Residual deviance:  96710  on 318945  degrees of freedom
  (81924 observations deleted due to missingness)
AIC: 96738

Number of Fisher Scoring iterations: 7
```

The score is not significant anymore, suggesting a cross-talk effect between highly correlated predictors. This is confirmed when the second sparsest score (1.97x10-7), which was previously moderately associated, is included in the analysis:

```
Call:
glm(formula = Phecode_411.8 ~ ., family = "binomial", data = d)

Deviance Residuals:
    Min       1Q   Median       3Q      Max
-1.2706  -0.3186  -0.2119  -0.1363   3.6533

Coefficients:
                                      Estimate Std. Error  z value Pr(>|z|)
(Intercept)                         -1.119e+01  1.088e-01 -102.895  < 2e-16 ***
Sex2                                -1.095e+00  2.046e-02  -53.513  < 2e-16 ***
Smoking_status1                      3.170e-01  1.993e-02   15.906  < 2e-16 ***
Smoking_status2                      6.112e-01  2.981e-02   20.503  < 2e-16 ***
Age_when_attended_assessment_centre  1.019e-01  1.476e-03   69.005  < 2e-16 ***
Qualifications2                      1.970e-01  3.092e-02    6.372 1.87e-10 ***
Qualifications3                      2.791e-01  2.407e-02   11.599  < 2e-16 ***
Qualifications4                      4.366e-01  4.166e-02   10.480  < 2e-16 ***
Qualifications5                      3.970e-01  2.982e-02   13.315  < 2e-16 ***
Qualifications6                      2.990e-01  3.477e-02    8.599  < 2e-16 ***
PC1                                  1.134e-03  2.057e-04    5.515 3.49e-08 ***
PC2                                 -4.709e-03  3.017e-04  -15.609  < 2e-16 ***
BMI                                  7.491e-02  1.785e-03   41.958  < 2e-16 ***
META.LRP11                          -5.957e-02  1.427e-01   -0.417    0.676
---
Signif. codes:  0 '***' 0.001 '**' 0.01 '*' 0.05 '.' 0.1 ' ' 1

(Dispersion parameter for binomial family taken to be 1)
```

```
    Null deviance: 109932  on 318958  degrees of freedom
Residual deviance:  96712  on 318945  degrees of freedom
  (81924 observations deleted due to missingness)
AIC: 96740

Number of Fisher Scoring iterations: 7
```

Together, these results suggest the association between LRP11 levels and PheCode 411.8 is spurious.

PILRB

Similarly, the single-score model of Type-2 diabetes using PILRB protein levels does not produce a significant predictor effect:

```
Call:
glm(formula = Phecode_250.2 ~ ., family = "binomial", data = d)

Deviance Residuals: 
    Min       1Q   Median       3Q      Max  
-2.5558  -0.3645  -0.2509  -0.1673   3.5155  

Coefficients:
                                  Estimate Std. Error  z value Pr(>|z|)    
(Intercept)                     -1.121e+01  8.560e-02 -130.921  < 2e-16 ***
Sex2                            -7.215e-01  1.658e-02  -43.529  < 2e-16 ***
Smoking_status1                  2.019e-01  1.716e-02   11.767  < 2e-16 ***
Smoking_status2                  4.497e-01  2.616e-02   17.193  < 2e-16 ***
Age_when_attended_assessment_centre  6.550e-02  1.136e-03   57.650  < 2e-16 ***
Qualifications2                  1.631e-01  2.639e-02    6.179 6.43e-10 ***
Qualifications3                  2.369e-01  2.061e-02   11.494  < 2e-16 ***
Qualifications4                  3.628e-01  3.322e-02   10.921  < 2e-16 ***
Qualifications5                  3.078e-01  2.662e-02   11.563  < 2e-16 ***
Qualifications6                  2.269e-01  3.074e-02    7.383 1.55e-13 ***
PC1                              3.908e-03  1.105e-04   35.355  < 2e-16 ***
PC2                             -7.320e-03  1.999e-04  -36.611  < 2e-16 ***
BMI                              1.613e-01  1.409e-03  114.483  < 2e-16 ***
META.PILRB.18                    5.788e-01  2.652e-01    2.182   0.0291 *  
---
Signif. codes:  0 ‘***’ 0.001 ‘**’ 0.01 ‘*’ 0.05 ‘.’ 0.1 ‘ ’ 1

(Dispersion parameter for binomial family taken to be 1)

    Null deviance: 144244  on 318958  degrees of freedom
Residual deviance: 123188  on 318945  degrees of freedom
  (81924 observations deleted due to missingness)
AIC: 123216

Number of Fisher Scoring iterations: 6
```

We iteratively repeat this model with all the 43 thresholded scores. None of the p-values of predictor association pass the Bonferroni-corrected mark of 0.05/43, making the association between PheCode 250.2 and predicted PILRB levels spurious.

MEP1B

A MEP1B score is significant for two different PheCodes: 250.2 and 411.4. We examine the first association, applying an iterative procedure as above. We similarly find that none of the individual scores are significant in the analysis. We extrapolate that the association with PheCode 411.4 is similarly spurious.

TNFRSF10C

We observe the same as above for the association between TNFRSF10C predicted levels and PheCode 250.2. Every individual score regression fails to reach significance, making this association spurious.

Supplementary Note 6: Sensitivity analysis

We repeat the analysis above by excluding related individuals as provided by the UK Biobank. The total sample size after exclusion is 407,146.

Model of PECAM1, CHI3L1, GRN excluding the ABO and CELSR2 regions

```
Call:
glm(formula = "high_cholesterol~.", family = binomial(link = "logit"),
    data = m)

Deviance Residuals:
    Min       1Q   Median       3Q      Max
-1.6176  -0.5377  -0.3842  -0.2577   3.1207

Coefficients:
                                  Estimate Std. Error  z value Pr(>|z|)
(Intercept)                     -9.1240558  0.0626596 -145.613  < 2e-16 ***
Sex2                            -0.5122541  0.0117205  -43.706  < 2e-16 ***
Smoking_status1                  0.1429592  0.0123099   11.613  < 2e-16 ***
Smoking_status2                  0.2267507  0.0202419   11.202  < 2e-16 ***
Age_when_attended_assessment_centre  0.0897144  0.0008468  105.948  < 2e-16 ***
Qualifications2                  0.0979410  0.0185111    5.291 1.22e-07 ***
Qualifications3                  0.1616728  0.0147011   10.997  < 2e-16 ***
Qualifications4                  0.2251346  0.0262725    8.569  < 2e-16 ***
Qualifications5                  0.1887324  0.0204498    9.229  < 2e-16 ***
Qualifications6                  0.1736789  0.0223700    7.764 8.23e-15 ***
PC1                              0.0011368  0.0001073   10.597  < 2e-16 ***
PC2                             -0.0034868  0.0001975  -17.659  < 2e-16 ***
PC3                              0.0055191  0.0003528   15.646  < 2e-16 ***
PC4                             -0.0023996  0.0004799   -5.000 5.72e-07 ***
PC5                             -0.0003605  0.0007788   -0.463   0.6434
PC6                             -0.0001831  0.0011116   -0.165   0.8692
PC7                              0.0001767  0.0010814    0.163   0.8702
PC8                             -0.0020898  0.0011460   -1.823   0.0682 .
PC9                              0.0005444  0.0012339    0.441   0.6591
PC10                             0.0056222  0.0012575    4.471 7.79e-06 ***
BMI                              0.0710085  0.0011431   62.120  < 2e-16 ***
PECAM1                          -0.0044688  0.0833686   -0.054   0.9573
CHI3L1                           0.6431507  0.1287930    4.994 5.92e-07 ***
GRN                              0.0700688  0.0639749    1.095   0.2734
---
Signif. codes:  0 '***' 0.001 '**' 0.01 '*' 0.05 '.' 0.1 ' ' 1

(Dispersion parameter for binomial family taken to be 1)

    Null deviance: 233657  on 332733  degrees of freedom
Residual deviance: 211942  on 332710  degrees of freedom
  (154675 observations deleted due to missingness)
AIC: 211990

Number of Fisher Scoring iterations: 5
```

AUC=0.7275

Model excluding GRN and PECAM1

```
Call:
glm(formula = "high_cholesterol~.", family = binomial(link = "logit"),
    data = m)

Deviance Residuals:
    Min       1Q   Median       3Q      Max
-1.6197  -0.5378  -0.3843  -0.2577   3.1225

Coefficients:
                                  Estimate Std. Error  z value Pr(>|z|)
```

```
(Intercept)                          -9.1293365  0.0622731 -146.602  < 2e-16 ***
Sex2                                 -0.5123250  0.0117203  -43.713  < 2e-16 ***
Smoking_status1                       0.1429393  0.0123098   11.612  < 2e-16 ***
Smoking_status2                       0.2266866  0.0202417   11.199  < 2e-16 ***
Age_when_attended_assessment_centre   0.0897152  0.0008468  105.949  < 2e-16 ***
Qualifications2                       0.0980315  0.0185108    5.296 1.18e-07 ***
Qualifications3                       0.1616832  0.0147011   10.998  < 2e-16 ***
Qualifications4                       0.2251751  0.0262725    8.571  < 2e-16 ***
Qualifications5                       0.1886731  0.0204495    9.226  < 2e-16 ***
Qualifications6                       0.1737365  0.0223698    7.767 8.06e-15 ***
PC1                                   0.0011293  0.0001062   10.632  < 2e-16 ***
PC2                                  -0.0034811  0.0001973  -17.647  < 2e-16 ***
PC3                                   0.0055474  0.0003517   15.774  < 2e-16 ***
PC4                                  -0.0024109  0.0004798   -5.025 5.03e-07 ***
PC5                                  -0.0003643  0.0007787   -0.468   0.6399    
PC6                                  -0.0001747  0.0011117   -0.157   0.8751    
PC7                                   0.0001793  0.0010813    0.166   0.8683    
PC8                                  -0.0020678  0.0011459   -1.805   0.0711 .  
PC9                                   0.0005604  0.0012338    0.454   0.6497    
PC10                                  0.0056242  0.0012575    4.472 7.73e-06 ***
BMI                                   0.0710026  0.0011431   62.116  < 2e-16 ***
CHI3L1                                0.6436717  0.1287891    4.998 5.80e-07 ***
---
Signif. codes:  0 '***' 0.001 '**' 0.01 '*' 0.05 '.' 0.1 ' ' 1

(Dispersion parameter for binomial family taken to be 1)

    Null deviance: 233657  on 332733  degrees of freedom
Residual deviance: 211943  on 332712  degrees of freedom
  (154675 observations deleted due to missingness)
AIC: 211987

Number of Fisher Scoring iterations: 5
```

AUC=0.7275

Null model

```
Call:
glm(formula = "high_cholesterol~.", family = binomial(link = "logit"), 
    data = m)

Deviance Residuals: 
    Min       1Q   Median       3Q      Max  
-1.6347  -0.5378  -0.3844  -0.2578   3.1021  

Coefficients:
                                      Estimate Std. Error  z value Pr(>|z|)    
(Intercept)                          -9.1508990  0.0621337 -147.278  < 2e-16 ***
Sex2                                 -0.5122531  0.0117197  -43.709  < 2e-16 ***
Smoking_status1                       0.1430282  0.0123092   11.620  < 2e-16 ***
Smoking_status2                       0.2266271  0.0202413   11.196  < 2e-16 ***
Age_when_attended_assessment_centre   0.0897030  0.0008467  105.939  < 2e-16 ***
Qualifications2                       0.0979009  0.0185097    5.289 1.23e-07 ***
Qualifications3                       0.1615258  0.0147001   10.988  < 2e-16 ***
Qualifications4                       0.2248044  0.0262716    8.557  < 2e-16 ***
Qualifications5                       0.1886945  0.0204488    9.228  < 2e-16 ***
Qualifications6                       0.1736805  0.0223694    7.764 8.22e-15 ***
PC1                                   0.0011477  0.0001061   10.813  < 2e-16 ***
PC2                                  -0.0034971  0.0001972  -17.731  < 2e-16 ***
PC3                                   0.0055385  0.0003516   15.750  < 2e-16 ***
PC4                                  -0.0025120  0.0004793   -5.241 1.60e-07 ***
PC5                                  -0.0003712  0.0007788   -0.477   0.6336    
PC6                                  -0.0002339  0.0011122   -0.210   0.8335    
PC7                                   0.0001871  0.0010813    0.173   0.8627    
PC8                                  -0.0021597  0.0011459   -1.885   0.0595 .  
PC9                                   0.0005543  0.0012336    0.449   0.6532    
```

```
PC10                                 0.0056818  0.0012575    4.518 6.23e-06 ***
BMI                                  0.0709815  0.0011429   62.104  < 2e-16 ***
---
Signif. codes:  0 '***' 0.001 '**' 0.01 '*' 0.05 '.' 0.1 ' ' 1

(Dispersion parameter for binomial family taken to be 1)

    Null deviance: 233657  on 332733  degrees of freedom
Residual deviance: 211968  on 332713  degrees of freedom
  (154675 observations deleted due to missingness)
AIC: 212010

Number of Fisher Scoring iterations: 5
```

AUC=0.7273

Original model including all three protein scores without any regional exclusion

```
Call:
glm(formula = "high_cholesterol~.", family = binomial(link = "logit"),
    data = m)

Deviance Residuals:
    Min       1Q   Median       3Q      Max
-1.6367  -0.5364  -0.3829  -0.2559   3.1443

Coefficients:
                                      Estimate Std. Error  z value Pr(>|z|)
(Intercept)                         -9.086e+00  6.259e-02 -145.185  < 2e-16 ***
Sex2                                -5.131e-01  1.173e-02  -43.739  < 2e-16 ***
Smoking_status1                      1.444e-01  1.232e-02   11.718  < 2e-16 ***
Smoking_status2                      2.283e-01  2.026e-02   11.268  < 2e-16 ***
Age_when_attended_assessment_centre  8.987e-02  8.476e-04  106.030  < 2e-16 ***
Qualifications2                      9.770e-02  1.853e-02    5.273 1.34e-07 ***
Qualifications3                      1.619e-01  1.471e-02   11.000  < 2e-16 ***
Qualifications4                      2.229e-01  2.629e-02    8.475  < 2e-16 ***
Qualifications5                      1.877e-01  2.047e-02    9.170  < 2e-16 ***
Qualifications6                      1.726e-01  2.239e-02    7.707 1.29e-14 ***
PC1                                  1.650e-03  1.094e-04   15.076  < 2e-16 ***
PC2                                 -3.096e-03  1.983e-04  -15.614  < 2e-16 ***
PC3                                  5.782e-03  3.519e-04   16.430  < 2e-16 ***
PC4                                 -1.852e-03  4.809e-04   -3.851 0.000118 ***
PC5                                  2.052e-04  7.807e-04    0.263 0.792666
PC6                                 -7.359e-05  1.114e-03   -0.066 0.947352
PC7                                 -3.273e-05  1.083e-03   -0.030 0.975886
PC8                                 -2.066e-03  1.147e-03   -1.801 0.071745 .
PC9                                  8.267e-04  1.236e-03    0.669 0.503618
PC10                                 5.592e-03  1.259e-03    4.442 8.91e-06 ***
BMI                                  7.109e-02  1.144e-03   62.119  < 2e-16 ***
PECAM1                              -6.829e-01  8.556e-02   -7.982 1.44e-15 ***
CHI3L1                               6.432e-01  1.289e-01    4.990 6.03e-07 ***
GRN                                  7.562e-01  3.932e-02   19.232  < 2e-16 ***
---
Signif. codes:  0 '***' 0.001 '**' 0.01 '*' 0.05 '.' 0.1 ' ' 1

(Dispersion parameter for binomial family taken to be 1)

    Null deviance: 233657  on 332733  degrees of freedom
Residual deviance: 211501  on 332710  degrees of freedom
  (154675 observations deleted due to missingness)
AIC: 211549

Number of Fisher Scoring iterations: 5
```

AUC=0.7296

DeLong's P-value for comparison of the CHI3L1 only versus the null model is equal to 0.0136. For the three-protein model versus the full model, the P-value is 7.81×10^{-27}. The corresponding likelihood ratio test p-values are 5.55×10^{-9} and 5.59×10^{-101}, respectively.

Supplementary Note 7: Coefficients of the elastic net model

The coefficients for the elastic net model described in the text, fit on the entire dataset for more accurate parameter estimation, are given in the table below. The qualifications variables are a dummy coding of the UK Biobank field 6138 using data coding 100305 where variable levels have been turned into labels, and the smoking status variables are a dummy coding of the UK Biobank field 20116 using data coding 90. For protein genetic risk scores, the P-value threshold is included in parentheses.

Variable group	Variable	Effect
	(Intercept)	0.587814192600955
	Sex	-0.0413162080627943
Smoking status (dummy variables, vs. never smoked)	Previous Smoker	0.0118989443411119
	Current Smoker	0.0111962448743365
	Age	0.00684558775742107
Qualifications (dummy vaiables, vs. University degree)	O levels/GCSEs or equivalent	0.00394959865872145
	NVQ or HND or HNC or equivalent	0.00895163532749535
	Other professional qualifications eg: nursing, teaching	0.00528342948315935
Principal components (PCs)	PC1	7.47342507105865e-05
	PC2	-0.000213788142332847
	PC3	0.00044419244443404
	PC10	3.83494557705365e-05
	BMI	0.00606310791867212
Protein scores	CHI3L1 ($P<9.25\text{x}10^{-7}$)	0.0199821126019553
	GRN ($P<7.45\text{x}10^{-11}$)	0.0453636405134527
	GRN ($P<4.57\text{x}10^{-9}$)	0.00589023195891766
	PECAM1 ($P<1.95\text{x}10^{-7}$)	-0.01608456535475
	SELE ($P<1.37\text{x}10^{-9}$)	-0.00651275802010374
	SELE ($P<8.16\text{x}10^{-8}$)	-0.00011048595528366
	SELE ($P<8.87\text{x}10^{-8}$)	-0.0174515150666494
	SELE ($P<1.77\text{x}10^{-7}$)	-0.00352572640455521

References

1. Yang, J., Ferreira, T., Morris, A.P., Medland, S.E., Genetic Investigation of, A.T.C., Replication, D.I.G., Meta-analysis, C., Madden, P.A., Heath, A.C., Martin, N.G., Montgomery, G.W., Weedon, M.N., Loos, R.J., Frayling, T.M., McCarthy, M.I., Hirschhorn, J.N., Goddard, M.E. & Visscher, P.M. Conditional and joint multiple-SNP analysis of GWAS summary statistics identifies additional variants influencing complex traits. *Nat Genet* **44**, 369-75, S1-3 (2012).
2. Groot, H.E., Villegas Sierra, L.E., Said, M.A., Lipsic, E., Karper, J.C. & van der Harst, P. Genetically Determined ABO Blood Group and its Associations With Health and Disease. *Arterioscler Thromb Vasc Biol* **40**, 830-838 (2020).
3. Klarin, D., Damrauer, S.M., Cho, K., Sun, Y.V., Teslovich, T.M., Honerlaw, J., Gagnon, D.R., DuVall, S.L., Li, J., Peloso, G.M., Chaffin, M., Small, A.M., Huang, J., Tang, H., Lynch, J.A., Ho, Y.L., Liu, D.J., Emdin, C.A., Li, A.H., Huffman, J.E., Lee, J.S., Natarajan, P., Chowdhury, R., Saleheen, D., Vujkovic, M., Baras, A., Pyarajan, S., Di Angelantonio, E., Neale, B.M., Naheed, A., Khera, A.V., Danesh, J., Chang, K.M., Abecasis, G., Willer, C., Dewey, F.E., Carey, D.J., Global Lipids Genetics, C., Myocardial Infarction Genetics, C., Geisinger-Regeneron Discov, E.H.R.C., Program, V.A.M.V., Concato, J., Gaziano, J.M., O'Donnell, C.J., Tsao, P.S., Kathiresan,

S., Rader, D.J., Wilson, P.W.F. & Assimes, T.L. Genetics of blood lipids among ~300,000 multi-ethnic participants of the Million Veteran Program. *Nat Genet* **50**, 1514-1523 (2018).
4. Zhou, D.H., Yang, L.N., Roder, C., Kalthoff, H. & Trauzold, A. TRAIL-induced expression of uPA and IL-8 strongly enhanced by overexpression of TRAF2 and Bcl-xL in pancreatic ductal adenocarcinoma cells. *Hepatobiliary Pancreat Dis Int* **12**, 94-8 (2013).
5. Briassouli, P., Komissarova, E.V., Clancy, R.M. & Buyon, J.P. Role of the urokinase plasminogen activator receptor in mediating impaired efferocytosis of anti-SSA/Ro-bound apoptotic cardiocytes: Implications in the pathogenesis of congenital heart block. *Circ Res* **107**, 374-87 (2010).
6. Ahola-Olli, A.V., Wurtz, P., Havulinna, A.S., Aalto, K., Pitkanen, N., Lehtimaki, T., Kahonen, M., Lyytikainen, L.P., Raitoharju, E., Seppala, I., Sarin, A.P., Ripatti, S., Palotie, A., Perola, M., Viikari, J.S., Jalkanen, S., Maksimow, M., Salomaa, V., Salmi, M., Kettunen, J. & Raitakari, O.T. Genome-wide Association Study Identifies 27 Loci Influencing Concentrations of Circulating Cytokines and Growth Factors. *Am J Hum Genet* **100**, 40-50 (2017).
7. Zernecke, A. & Weber, C. Chemokines in atherosclerosis: proceedings resumed. *Arterioscler Thromb Vasc Biol* **34**, 742-50 (2014).
8. Wan, W., Liu, Q., Lionakis, M.S., Marino, A.P., Anderson, S.A., Swamydas, M. & Murphy, P.M. Atypical chemokine receptor 1 deficiency reduces atherogenesis in ApoE-knockout mice. *Cardiovasc Res* **106**, 478-87 (2015).
9. GTEx Consortium. Human genomics. The Genotype-Tissue Expression (GTEx) pilot analysis: multitissue gene regulation in humans. *Science* **348**, 648-60 (2015).
10. Sivagnanam, M., Mueller, J.L., Lee, H., Chen, Z., Nelson, S.F., Turner, D., Zlotkin, S.H., Pencharz, P.B., Ngan, B.Y., Libiger, O., Schork, N.J., Lavine, J.E., Taylor, S., Newbury, R.O., Kolodner, R.D. & Hoffman, H.M. Identification of EpCAM as the gene for congenital tufting enteropathy. *Gastroenterology* **135**, 429-37 (2008).
11. Stoka, V., Turk, V. & Turk, B. Lysosomal cathepsins and their regulation in aging and neurodegeneration. *Ageing Res Rev* **32**, 22-37 (2016).
12. Carithers, L.J., Ardlie, K., Barcus, M., Branton, P.A., Britton, A., Buia, S.A., Compton, C.C., DeLuca, D.S., Peter-Demchok, J., Gelfand, E.T., Guan, P., Korzeniewski, G.E., Lockhart, N.C., Rabiner, C.A., Rao, A.K., Robinson, K.L., Roche, N.V., Sawyer, S.J., Segre, A.V., Shive, C.E., Smith, A.M., Sobin, L.H., Undale, A.H., Valentino, K.M., Vaught, J., Young, T.R., Moore, H.M. & Consortium, G.T. A Novel Approach to High-Quality Postmortem Tissue Procurement: The GTEx Project. *Biopreserv Biobank* **13**, 311-9 (2015).
13. Tachmazidou, I., Hatzikotoulas, K., Southam, L., Esparza-Gordillo, J., Haberland, V., Zheng, J., Johnson, T., Koprulu, M., Zengini, E., Steinberg, J., Wilkinson, J.M., Bhatnagar, S., Hoffman, J.D., Buchan, N., Suveges, D., arc, O.C., Yerges-Armstrong, L., Smith, G.D., Gaunt, T.R., Scott, R.A., McCarthy, L.C. & Zeggini, E. Identification of new therapeutic targets for osteoarthritis through genome-wide analyses of UK Biobank data. *Nat Genet* **51**, 230-236 (2019).
14. Sun, B.B., Maranville, J.C., Peters, J.E., Stacey, D., Staley, J.R., Blackshaw, J., Burgess, S., Jiang, T., Paige, E., Surendran, P., Oliver-Williams, C., Kamat, M.A., Prins, B.P., Wilcox, S.K., Zimmerman, E.S., Chi, A., Bansal, N., Spain, S.L., Wood, A.M., Morrell, N.W., Bradley, J.R., Janjic, N., Roberts, D.J., Ouwehand, W.H., Todd, J.A., Soranzo, N., Suhre, K., Paul, D.S., Fox, C.S., Plenge, R.M., Danesh, J., Runz, H. & Butterworth, A.S. Genomic atlas of the human plasma proteome. *Nature* **558**, 73-79 (2018).
15. Kamat, M.A., Blackshaw, J.A., Young, R., Surendran, P., Burgess, S., Danesh, J., Butterworth, A.S. & Staley, J.R. PhenoScanner V2: an expanded tool for searching human genotype-phenotype associations. *Bioinformatics* 10.1093/bioinformatics/btz469(2019).
16. Koscielny, G., An, P., Carvalho-Silva, D., Cham, J.A., Fumis, L., Gasparyan, R., Hasan, S., Karamanis, N., Maguire, M., Papa, E., Pierleoni, A., Pignatelli, M., Platt, T., Rowland, F., Wankar, P., Bento, A.P., Burdett, T., Fabregat, A., Forbes, S., Gaulton, A., Gonzalez, C.Y., Hermjakob, H., Hersey, A., Jupe, S., Kafkas, S., Keays, M., Leroy, C., Lopez, F.J., Magarinos, M.P., Malone, J., McEntyre, J., Munoz-Pomer Fuentes, A., O'Donovan, C., Papatheodorou, I., Parkinson, H., Palka, B., Paschall, J., Petryszak, R., Pratanwanich, N., Sarntivijal, S., Saunders,

G., Sidiropoulos, K., Smith, T., Sondka, Z., Stegle, O., Tang, Y.A., Turner, E., Vaughan, B., Vrousgou, O., Watkins, X., Martin, M.J., Sanseau, P., Vamathevan, J., Birney, E., Barrett, J. & Dunham, I. Open Targets: a platform for therapeutic target identification and validation. *Nucleic Acids Res* **45**, D985-D994 (2017).

17. Wishart, D.S., Knox, C., Guo, A.C., Cheng, D., Shrivastava, S., Tzur, D., Gautam, B. & Hassanali, M. DrugBank: a knowledgebase for drugs, drug actions and drug targets. *Nucleic Acids Res* **36**, D901-6 (2008).
