# Supplementary material for: Whole-genome sequencing analysis of the cardiometabolic proteome
Source: Nat Commun. 2020 Dec 10;11:6336. doi: 10.1038/s41467-020-20079-2 (PMC7729872; doi:10.1038/s41467-020-20079-2)
Supplement: Supplementary file 3 — Description of Additional Supplementary Files [file 41467_2020_20079_MOESM3_ESM.pdf]

Supplementary Data Legends

Supplementary Data 1. a: Study-wide significant cis-pQTL. b: Study-wide significant trans pQTL.

**column**	**description**
**panel**	OLINK protein panel on which the protein was assayed
**protein**	protein name as provided by OLINK
**lead SNP**	index SNP defining a 2Mb locus
**independent SNP**	independently associated SNP at locus, as defined by COJO
**novelty_status**	does the p-value of the independent SNP conditioned on all previous signals pass the study-wide significance threshold of 7.45e-11
**reference MANOLIS**	reference allele GRCh38 in discovery
**alternate MANOLIS**	alternate allele GRCh38 in discovery
**AF MANOLIS**	alternate allele frequency in discovery
**effect allele MANOLIS**	effect allele
**MAF MANOLIS**	minor allele frequency in discovery
**beta MANOLIS**	discovery effect size
**se MANOLIS**	discovery standard error of the effect size
**p MANOLIS**	score test discovery p-value
**reference Pomak**	reference allele GRCh38 in replication
**alternate Pomak**	alternate allele GRCh38 in replication
**AF Pomak**	allele frequency in replication
**effect allele Pomak**	effect allele in replication
**MAF Pomak**	minor allele frequency in replication
**beta Pomak**	effect size in replication
**se Pomak**	standard error in replication
**p Pomak**	score test replication p-value
**proxy if not present in Pomak**	tagging variant (r2>0.8) used for meta-analysis if independent SNP not present in replication cohort
**beta**	meta-analysis effect size
**se**	meta-analysis standard error
**p**	inverse variance based meta-analysis p-value
**rsids**	Rsids of the discovery variant if present
**replicates**	does the meta-analysis p-value pass the Bonferroni-corrected threshold of nominal significance
**cis consequence**	consequence on the *cis* gene as per Ensembl VEP

**cis gene ensembl id**	Ensembl ID of the *cis* gene
**distance to cis (bp)**	distance to the above, as per Ensembl REST API
**cis gene name**	Gene symbol, as per REST API
**uniprot id**	UniProt protein name
**uniprot short name**	UniProt short name
**uniprot long name**	UniProt long name
**P conditional**	maximum conditional score test p-value across all previously associated variants in the region for the same protein. "same variant" indicates the variant itself is known.
**not studied before**	literature search and GWAS catalog did not produce evidence of previous genetic associations for this protein
**novel locus**	have there never been any reported associations for this protein at this locus
**mapped gene consequence**	for *trans* loci, consequence of the discovery variant on the mapped gene
**mapped gene ID**	for *trans* loci, Ensemb ID of the mapped gene
**mapped gene distance (bp)**	for *trans* loci, distance to the mapped gene as per REST API
**mapped gene**	for *trans* loci, gene that the signal has been mapped to. Details on how this is done are provided in the Methods section of the paper.
**receptor/ligand**	does the association reflect a documented receptor/ligand pair

Supplementary Data 2: Allele frequencies in MANOLIS compared to reference European populations. Alternate allele counts according to GRCh38 are compared to an absolute population alternate frequency. If gnomAD non-Finnish European counts are available for exomes, those are used for comparison. If they are not, gnomAD non-Finnish European genomes are used. If the variant is not present in gnomAD, TOPMed frequencies are used as reference.

Supplementary Data 3. a: Phenotypes used for 2-sample MR in MRBase. All the information displayed in this table is a filtered output from the MRBase get_available_outcomes function. "id" is the MRBase ID. b: Phenotypes used for 2-sample MR using manually downloaded statistics. For every study, we display the file name used, sample sizes broken down by cases and controls, a description of the trait and a PMID for the supporting paper.

Supplementary Data 4: 2-Sample MR results. Exposures are reported according to their OLINK panel name and OLINK protein name separated by a dot. The method is Wald ratio for single SNP instruments and Inverse-variance weighted for multi-SNV instruments. The FDR-corrected p-value is displayed in the pBH column, which is filtered for significant results (pBH<0.05). Outcome names are as given by MR-Base for that trait, or equal to the filenames used in case of manually downloaded summary statistics, as described in Supplementary Data 3b.

Supplementary Data 5: Directions of effect for outcomes and instruments in Mendelian randomization analysis. In case a pQTL locus included in MR involved variants with both positive and negative effects, their direction is denoted as "mixed".

Supplementary Data 6. a: Effect of protein level genetic scores in logistic disease prediction models in UK Biobank, for most significantly associated protein scores. Effect: log-odds change per score unit increase β : effect size, σ: standard error. Wald P: P-value for the Wald test of predictor significance. Threshold: P threshold used for constructing the score. SR: Self-reported. ICD: International Classification of Diseases, Tenth Revision. b: Logistic regression models of disease states on protein PRS and clinical factors. When several scores for the same protein pass the Bonferroni-corrected p-value threshold, only the model with the strongest evidence of score effects across all p-value thresholds is reported. Only models where a protein score passes the significance threshold of 0.05/(47*86) are reported here.

Supplementary Data 7: Proteins measured in this study, and reasons for exclusion if applicable. The symbol column denotes the internal name provided by the vendor (Olink) used throughout this study.

Supplementary Data 8: Two-sample MR results for the PDL2 gene association, when only the cis locus is included as an instrument. Rows are sorted by ascending p-value. P-values are reported for the test specified in the "method" column (one-sided). Highlighted rows pass the FDR-adjusted significance threshold.

Supplementary Data 9: Variance explained by MANOLIS PRS in the Pomak cohort as a function of MAF and P-value thresholds for inclusion

Supplementary Data 10. a: Optimal score selection and R-squared for protein level prediction in Pomak, using summary statistics in MANOLIS using the full dataset. This analysis is a repeat of that presented in ST12 using a more recent version of PRSice (2.3.1.e) and is presented for comparison purposes only. b: Optimal score selection and R-squared for protein level prediction in Pomak, using summary statistics in MANOLIS using repeated cross-validation (repeats:3, folds:5). c: Optimal score selection and R-squared for protein level prediction in Pomak, using summary statistics in MANOLIS, using the full dataset, with a 2Mb region around ABO excluded from the analysis. Only the four proteins found to be correlated with each other and with evidence of a SNV in ABO driving the top score are considered here.

Supplementary Data 11. a: Non-cancer illness codes, with description and number of cases, used for polygenic prediction in UK Biobank. b: ICD10 codes (primary and secondary) with more than 999 cases used for polygenic prediction in UK Biobank.

Supplementary Data 12: Association between protein scores and PheCodes. The table, which reports all coefficients, is filtered for significant protein scores: the variable column should be a protein and the P-value column should be smaller than 0.05/(43*47), correcting for the number of proteins and PheCodes tested. Suffixes in the variable column correspond to P-value thresholds for building scores, 1 corresponds to the most stringent, at 7.45x10-11, with a step of 1x10-10. The null model is the model without any protein scores.

Supplementary Data 13: List of GWAS individual accession numbers used in this study.

Supplementary Data 14: Variants included in the burdens of the selected associations. Alleles: reference/non-reference alleles; Consequence: the most severe consequence predicted by Ensembl VEP; MAF: minor allele frequency; AC: non-reference allele count; Weight: the weight assigned to the variant in the most significant test as per Supplementary Data 15; NA indicates that no weight was applied.

Supplementary Data 15: Non-proteomic phenotypes used for comparison in the MANOLIS cohort. "rbin" indicates inverse normal transformation, ln indicates natural logarithm. The last three columns indicate whether the corresponding covariate was adjusted for.

Supplementary Data 16: PheWAS results for all independent variants reported in this study. P4 is the posterior probability of a single colocalising signal. Alpha12 and alpha21 are the products of the effect sizes for the protein and the tested traits, n is the number of variants input in the colocalisation test.

Supplementary Data 17: Extract of the DrugBank database where target genes correspond to cis genes for proteins measured in the current study

Supplementary Data 18: Mouse orthologs and their phenotype associations as provided by MGI for genes coding for the proteins included in our study. P-values are hard-filtered using Bonferroni correction for the number of orthologs tested.
